# Supplementary material for: Longitudinal changes in auditory and reward systems following receptive music-based intervention in older adults
Source: Sci Rep. 2022 Jul 7;12:11517. doi: 10.1038/s41598-022-15687-5 (PMC9261172; doi:10.1038/s41598-022-15687-5)
Supplement: Supplementary file 1 — Supplementary Information. [file 41598_2022_15687_MOESM1_ESM.docx]

# Supplementary Materials

for

Longitudinal Changes in Auditory and Reward Systems Following Receptive Music-Based Intervention in Older Adults

by

Milena Aiello Quinci^1^, Alexander Belden^1^, Valerie Goutama1, Dayang Gong^1^, Suzanne Hanser^2^, Nancy J. Donovan^3^, Maiya Geddes^3,4^, Psyche Loui^1*^

^1^ Northeastern University

^2^ Berklee College of Music

^3^ Brigham and Women’s Hospital and Harvard Medical School

^4^ McGill University

* Corresponding author: p.loui@northeastern.edu
360 Huntington Ave, ISEC 129, Boston MA 02115 USA

**Table S1** | Researcher-Selected Song List

|  |  |  | Title | Artist/Composer | Music Selection |
| --- | --- | --- | --- | --- | --- |
|  |  |  | Adagio for Strings | Barber | Well-Known |
|  |  |  | Symphony No. 2, Finale | Mahler | Well-Known |
|  |  |  | Cavatina Op. 130 | Beethoven | Well-Known |
|  |  |  | John Wayne Gacy Jr. | Sufjan Stevens | Well-Known |
|  |  |  | Hurt | Johnny Cash | Well-Known |
|  |  |  | Sound of Silence | Simon and Garfunkel | Well-Known |
|  |  |  | Reckoner | Radiohead | Well-Known |
|  |  |  | Nocturne in C Sharp Minor | Chopin | Well-Known |
|  |  |  | Nessun Dorma | Andrea Bocelli | Well-Known |
|  |  |  | Symphony No. 6, movement 1 | Tchaikovsky | Well-Known |
|  |  |  | Bohlen-Pierce Pan Flute Folk Tune | Arturo Grolimund | Novel |
|  |  |  | Hoquetus II | Johannes Kretz | Novel |
|  |  |  | Reminiscences | Steven Yi | Novel |
|  |  |  | Beyond The Horizon | Georg Hajdu | Novel |
|  |  |  | When the Moon Casts Shadows | Hubert Ho | Novel |
|  |  |  | Simmer | Hubert Ho | Novel |
|  |  |  | Vertigo Temporum | Hubert Ho | Novel |
|  |  |  | Manual Labor | Hubert Ho | Novel |
|  |  |  |  |  |  |

**
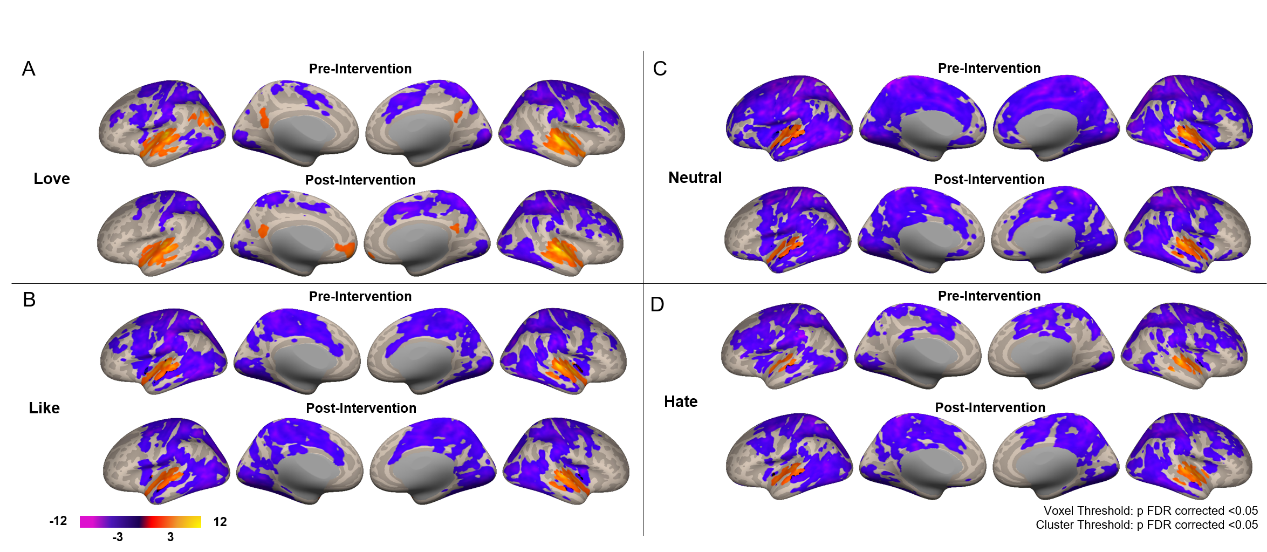
**

**Figure S1. Univariate whole-brain analyses for different levels of liking. (A) Love.** Auditory and DMN regions showed activity both pre- and post-intervention, with the TPJ showing pre-intervention activity only. Motor areas showed deactivation both pre- and post-intervention.  **(B) Like.** Auditory regions showed activity both pre- and post-intervention. Regions spanning the motor and DMN areas showed deactivation both pre- and post-intervention. **(C) Neutral.** Auditory regions showed activity both pre- and post-intervention. Regions spanning the motor and DMN areas showed deactivation both pre- and post-intervention. **(D) Hate.** Auditory regions showed activity both pre- and post-intervention. Regions spanning the motor and DMN areas showed deactivation both pre- and post-intervention. All images are results of second-level analyses showing significant clusters at the p < .05 FDR-corrected level.


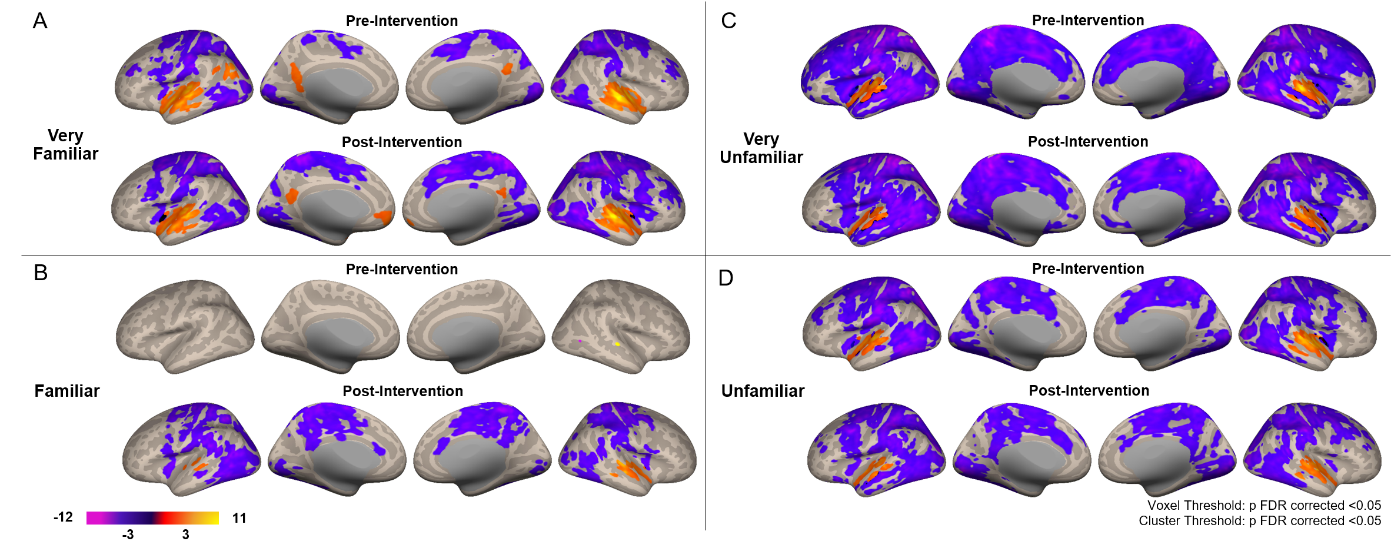


**Figure S2. Univariate whole-brain analyses for different levels of familiarity. (A) Very Familiar.** Auditory and DMN regions showed activity both pre- and post-intervention, with the TPJ showing pre-intervention activity only. Motor areas showed deactivation both pre- and post-intervention.  **(B) Familiar.** Auditory regions showed activity both pre- and post-intervention, post-intervention to a greater extent. Motor regions showed deactivation only for post-intervention. **(C) Very Unfamiliar.** Auditory regions showed activity both pre- and post-intervention. Regions spanning the motor and DMN areas showed deactivation both pre- and post-intervention. **(D) Unfamiliar.** Auditory regions showed activity both pre- and post-intervention. Regions spanning the motor and DMN areas showed deactivation both pre- and post-intervention. All images are results of second-level analyses showing significant clusters at the p < .05 FDR-corrected level.


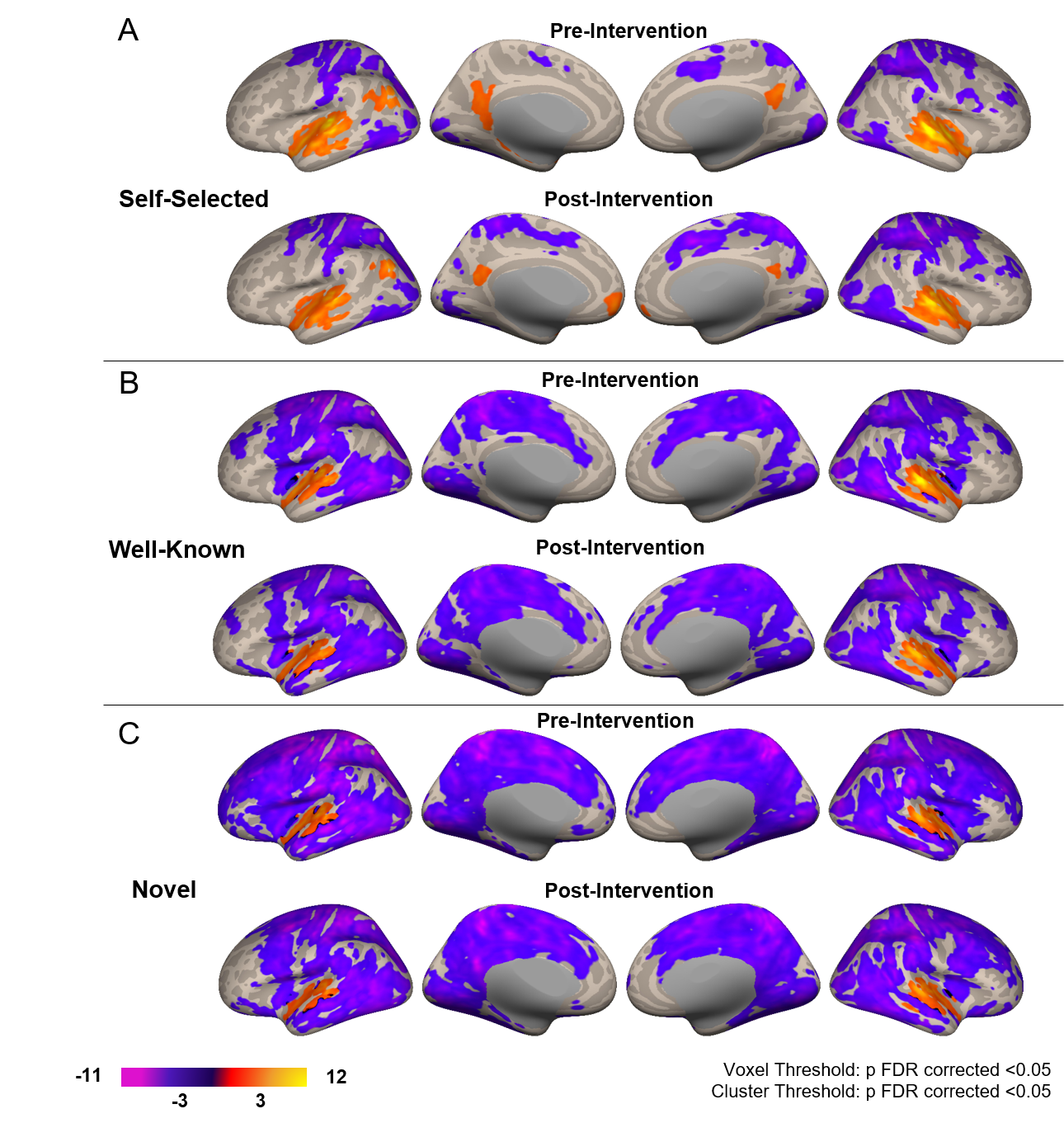


**Figure S3. Univariate whole-brain analyses for music selection. (A) Self-Selected.** Auditory and DMN regions showed activity both pre- and post-intervention. Motor areas showed deactivation both pre- and post-intervention.  **(B) Well-Known.** Auditory regions showed activity both pre- and post-intervention. Regions spanning the motor and DMN areas showed deactivation both pre- and post-intervention. **(C) Novel.** Auditory regions showed activity both pre- and post-intervention. Regions spanning the motor and DMN areas showed deactivation both pre- and post-intervention. All images are results of second-level analyses showing significant clusters at the p < .05 FDR-corrected level.


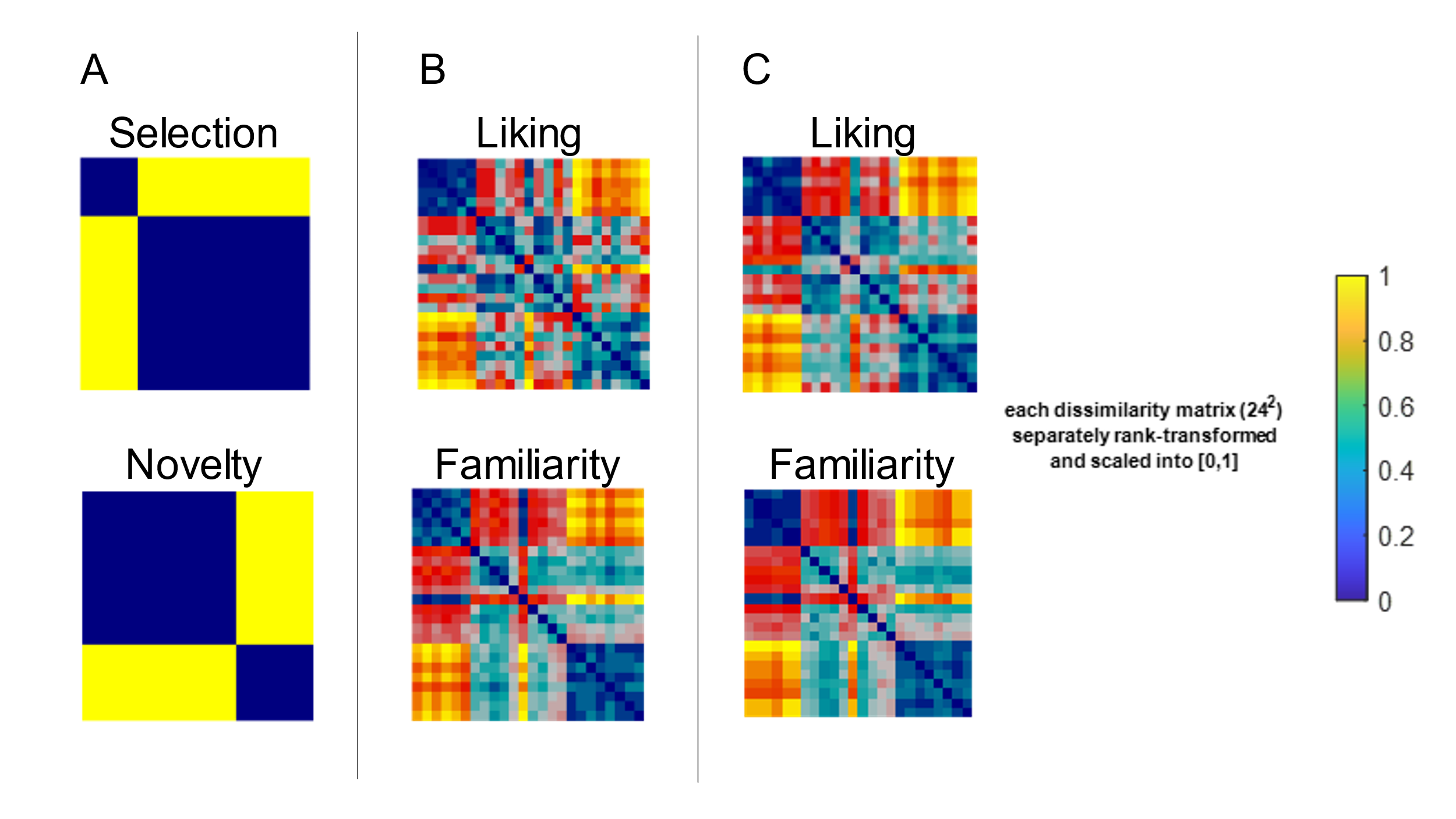


**Figure S4: Second Level Model RDMs.** (A) Time invariant binary RDMs representing stimulus selection (top) and novelty (bottom). (B) Participant-averaged RDMs representing stimulus liking (top) and familiarity (bottom) ratings at the pre-intervention timepoint. (C) Participant-averaged RDMs representing stimulus liking (top) and familiarity (bottom) ratings at the post-intervention timepoint.


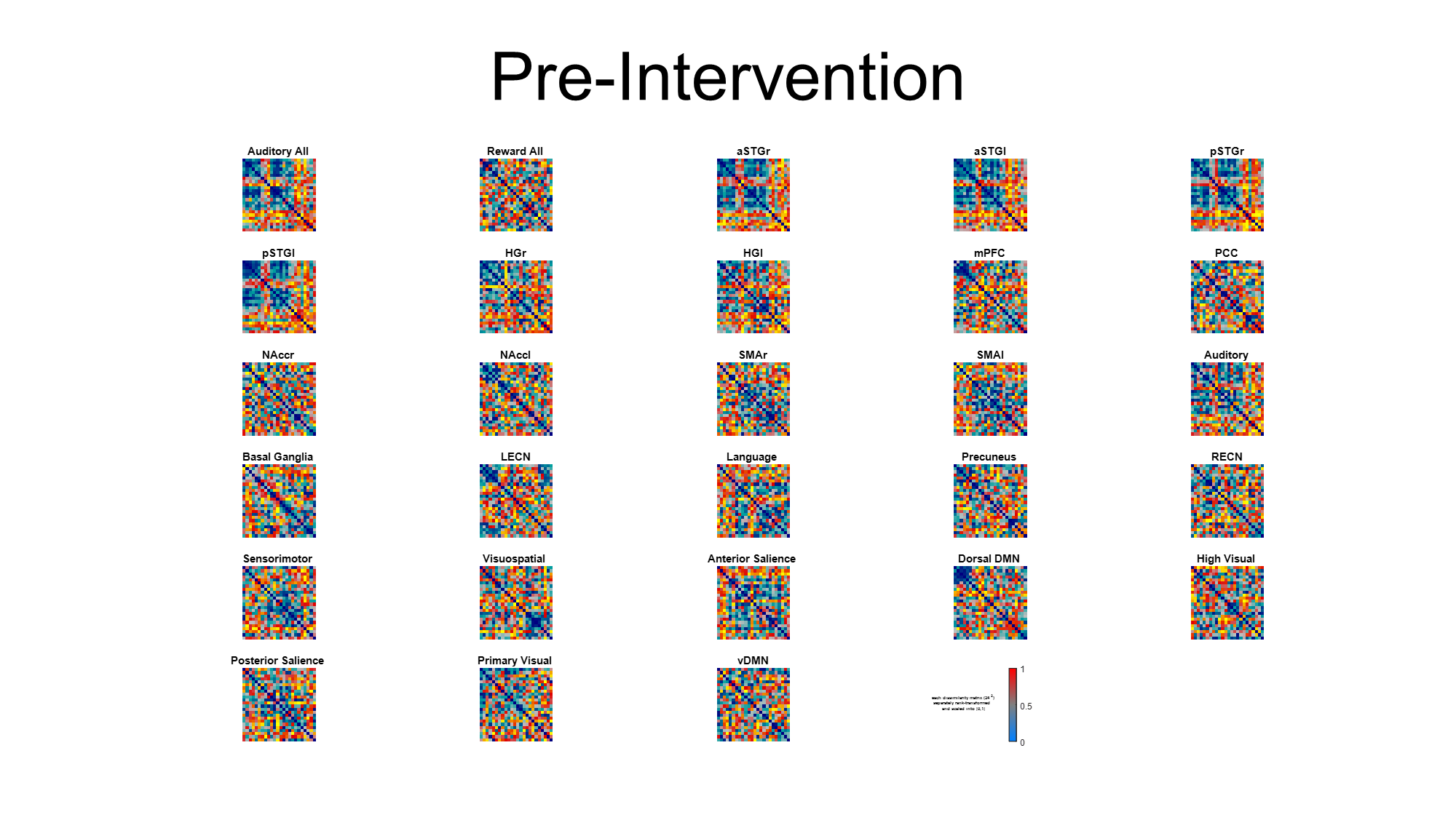


**Figure S5: Pre-intervention fMRI-Derived RDMs.** Participant-averaged RDMs representing differences in cortical responses to stimuli at the pre-intervention timepoint.


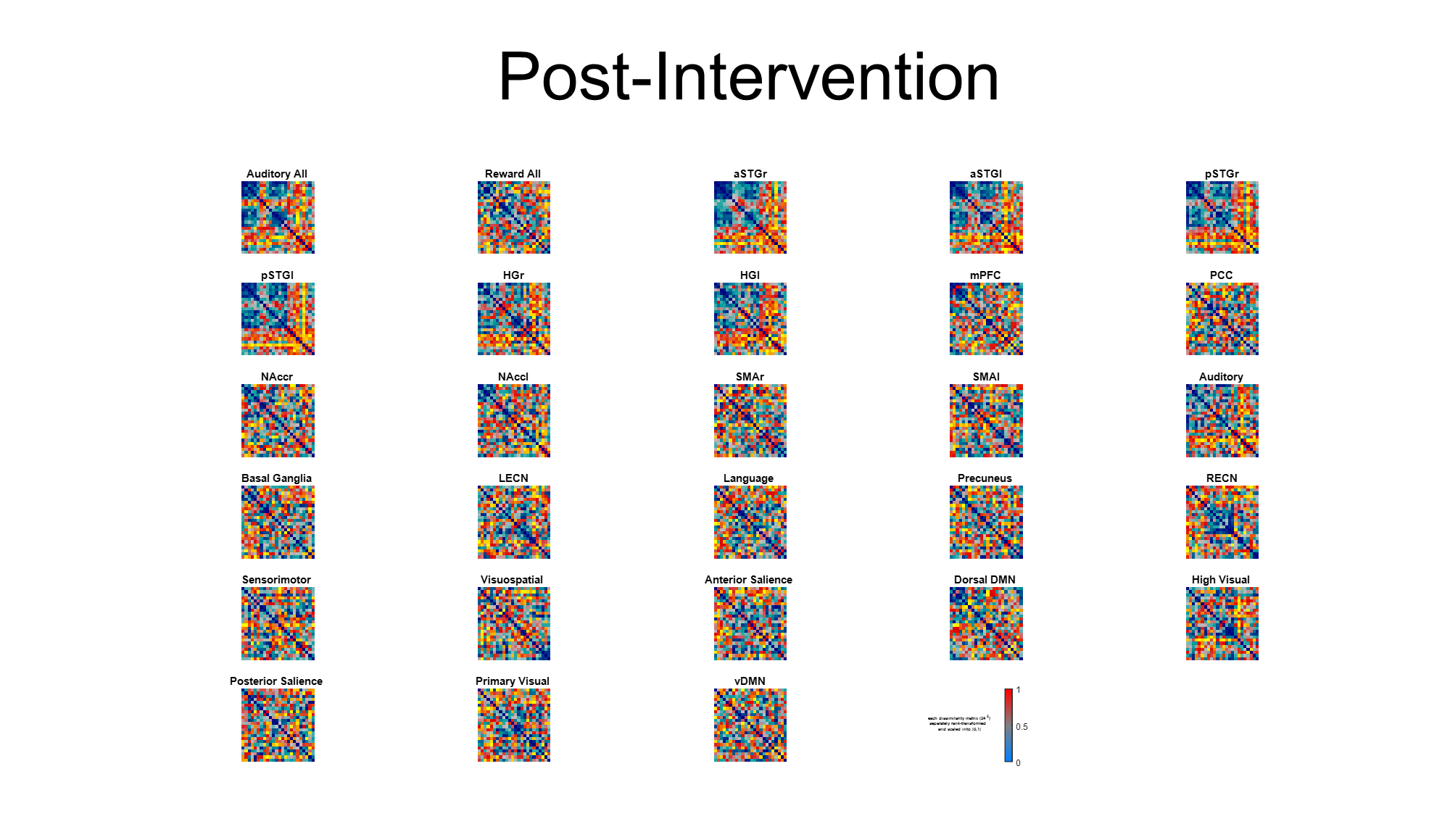


**Figure S6: Post-intervention fMRI-Derived RDMs.** Participant-averaged RDMs representing differences in cortical responses to stimuli at the post-intervention timepoint.


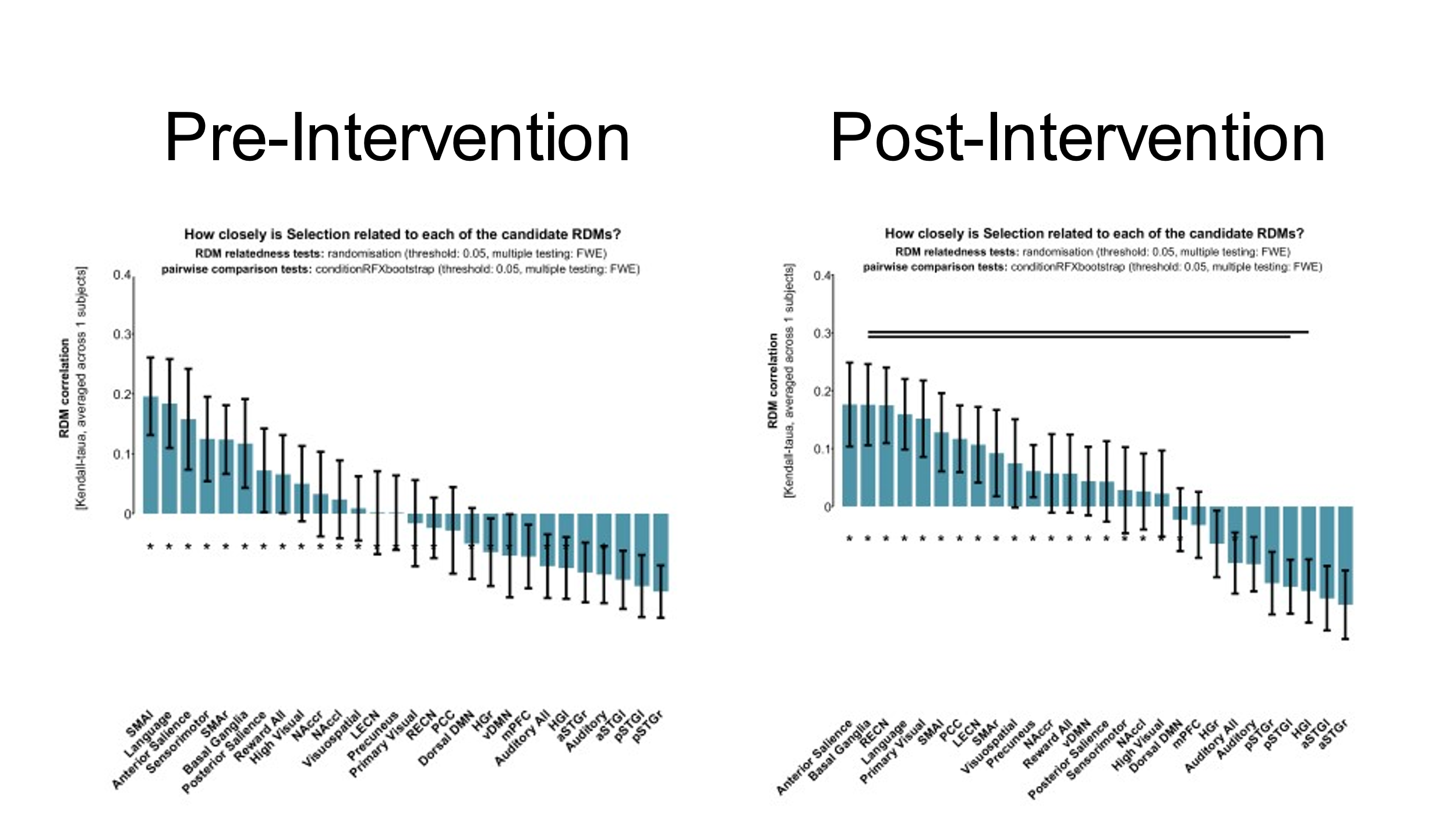


**Figure S7: Bootstrap Comparisons of Selection RDM.** results of 10,000-fold bootstrap comparisons of selection RDM to second level fMRI-derived RDMs at pre-intervention (left) and post-intervention (right) time points. Asterisk (*) represents a significant correlation between selection and a given fMRI-derived RDM (p <0.05, FWE corrected), and Black bars represent a significant difference between fMRI-derived RDMs (p <0.05, FWE corrected)


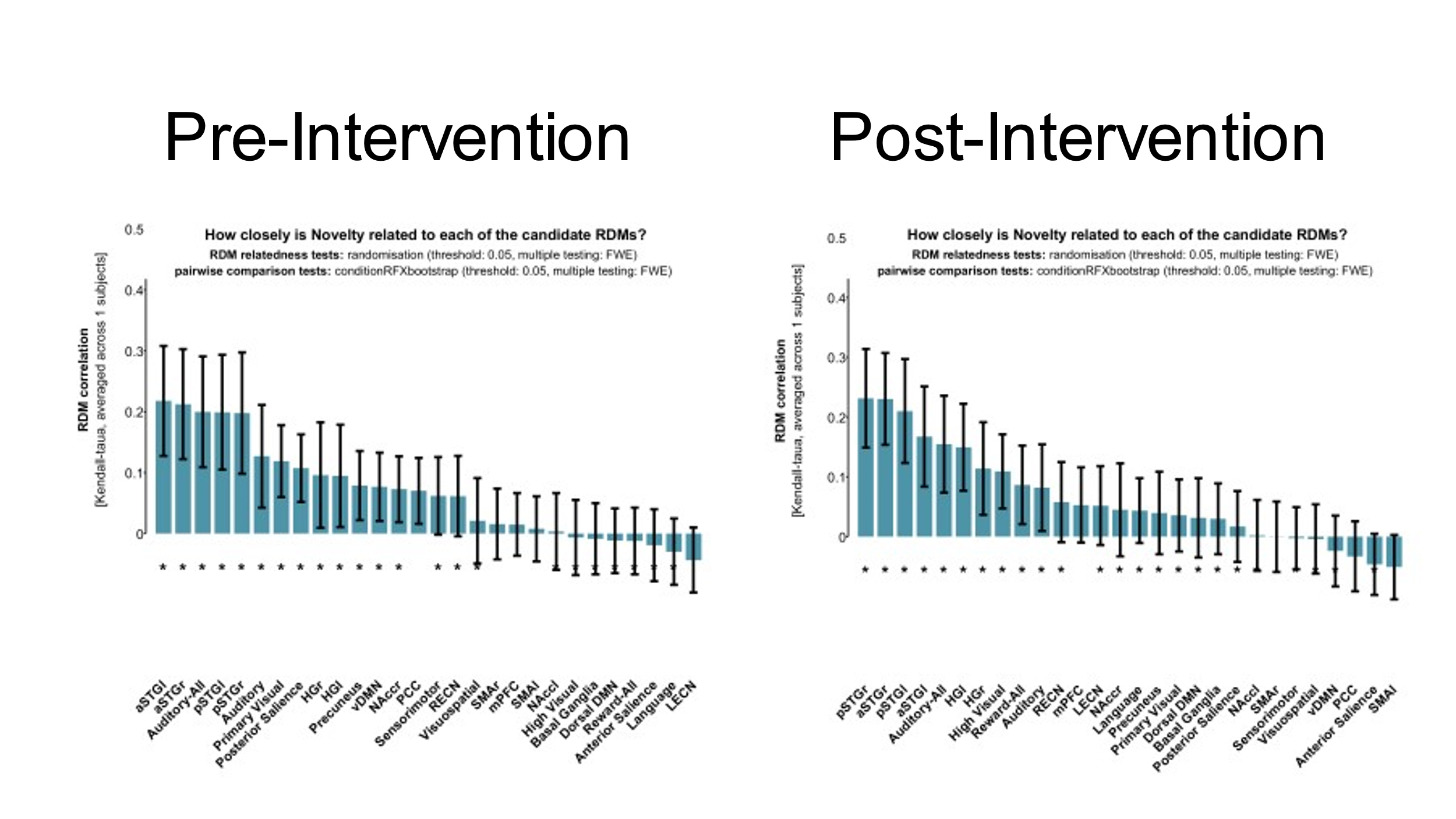


**Figure S8: Bootstrap Comparisons of Novelty RDM.** results of 10,000-fold bootstrap comparisons of novelty RDM to second level fMRI-derived RDMs at pre-intervention (left) and post-intervention (right) time points. Asterisk (*) represents a significant correlation between novelty and a given fMRI-derived RDM (p <0.05, FWE corrected).


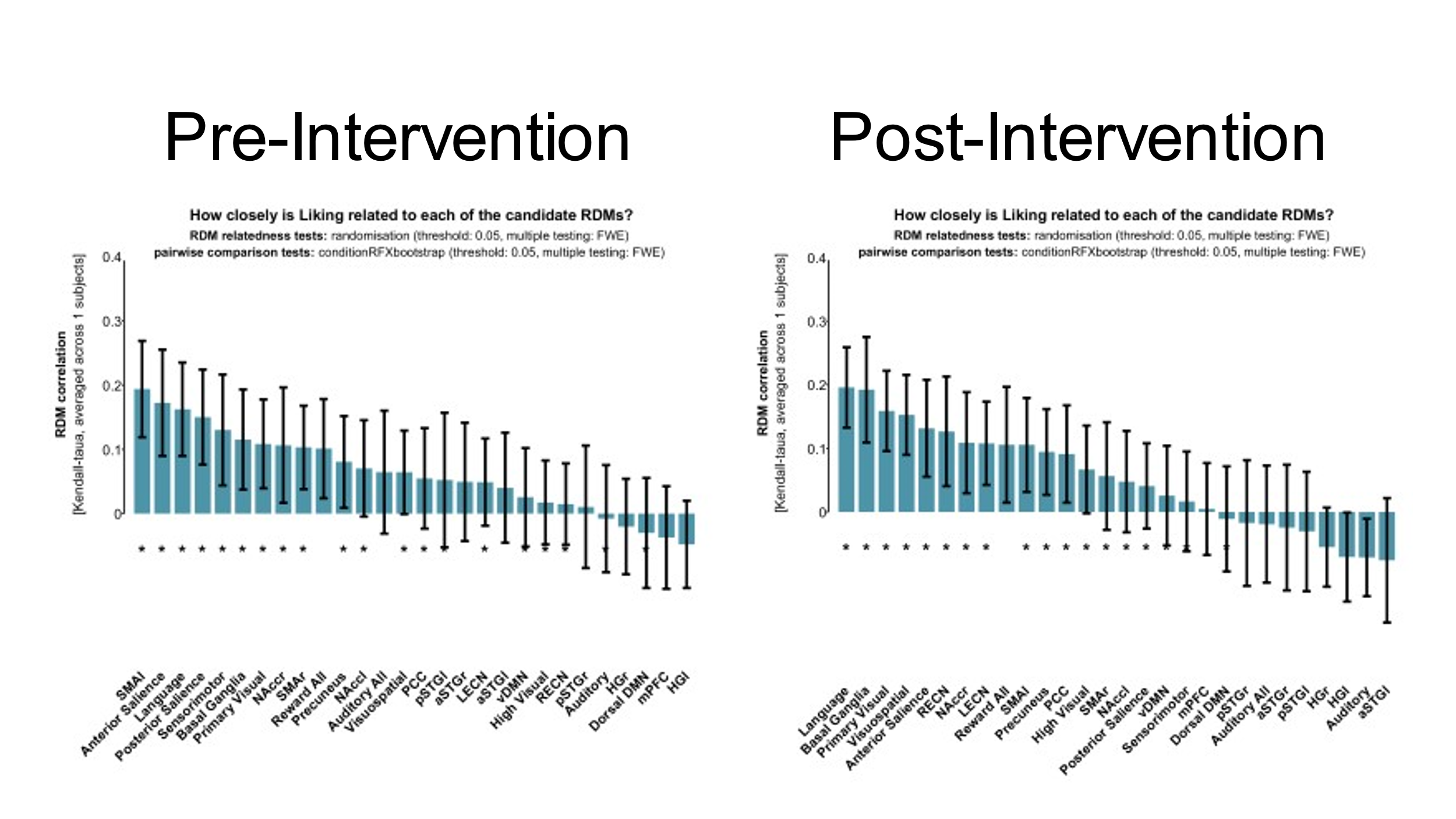


**Figure S9: Bootstrap Comparisons of Liking RDM.** results of 10,000-fold bootstrap comparisons of second-level liking RDM to second level fMRI-derived RDMs at pre-intervention (left) and post-intervention (right) time points. Asterisk (*) represents a significant correlation between liking and a given fMRI-derived RDM (p <0.05, FWE corrected).


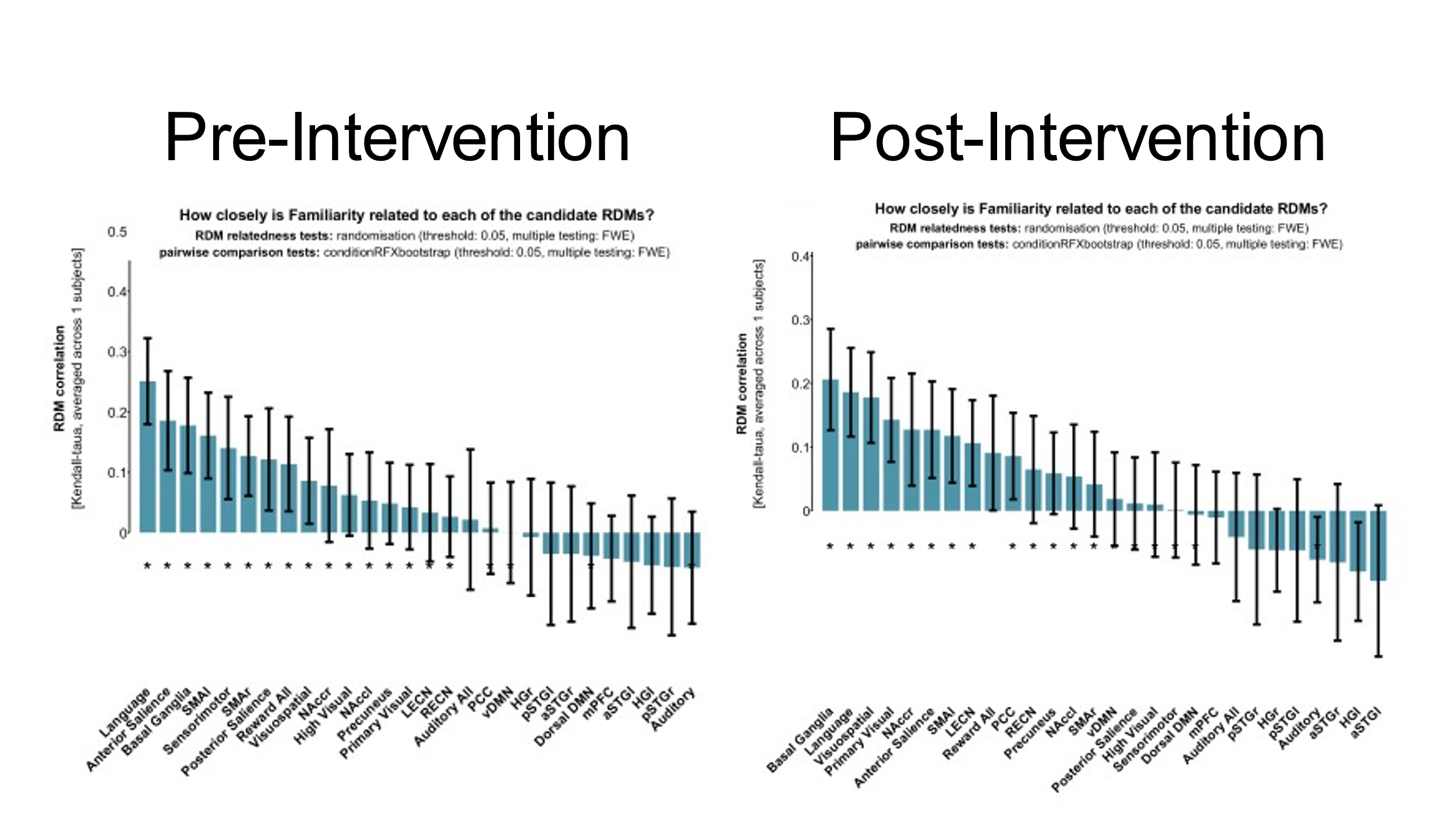


**Figure S10: Bootstrap Comparisons of Familiarity RDM.** results of 10,000-fold bootstrap comparisons of second-level familiarity RDM to second level fMRI-derived RDMs at pre-intervention (left) and post-intervention (right) time points. Asterisk (*) represents a significant correlation between liking and a given fMRI-derived RDM (p <0.05, FWE corrected).
